# Quantifying Dexterity and Grip Strength Associations between Age, Cognitive Function (MoCA), and Cerebellar Gray Matter Volume in Healthy Adults across the Adult Lifespan

**DOI:** 10.21203/rs.3.rs-10375298/v1

**Published:** 2026-07-23

**Authors:** Pranesh Rajesh Kannan, Raghav Pallapothu, Santosh Kudaravalli, Sriya Pallapothu, Roger D. Newman-Norlund

**Affiliations:** University of South Carolina; University of South Carolina; University of South Carolina; University of South Carolina; University of South Carolina

**Keywords:** Cerebellum, Crus II, Grip strength, Manual dexterity, Cognitive aging, Gray matter volume

## Abstract

**Introduction:**

Cerebellar Crus I/II gray matter volume (GMV) is a structural marker of cognitive aging, and motor performance (grip strength and manual dexterity) predicts age-related cognitive decline. Whether these motor measures dissociate in their cerebellar and cognitive correlates is unknown.

**Methods:**

We examined Pearson partial correlations (age, sex, and race covaried) between grip strength, Nine-Hole Peg Test (9HPT) dexterity, cerebellar Crus I/II GMV, and Montreal Cognitive Assessment (MoCA) subscores in 304 healthy adults aged 20–79 from the University of South Carolina Aging Brain Cohort. GMV was derived from T1-weighted magnetic resonance imaging using the Computational Anatomy Toolbox (CAT12) with the Automated Anatomical Labeling 3 (AAL3) atlas.

**Results:**

After false discovery rate (FDR) correction, dominant 9HPT time correlated with MoCA total score (*r*=−.240, *p*_FDR_<.001) and memory index (*r*=−.220, *p*_FDR_<.001); no other MoCA subscores survived. Grip strength was not associated with any MoCA measure but correlated bilaterally with Crus II GMV (*r*=.156–.224, all *p*_FDR_≤.038). Bootstrapped mediation (5,000 iterations) revealed that dexterity partially mediated age effects on cognition (28.8% mediated; 95% confidence interval [−0.022,−0.006]), and Crus II volume partially mediated age effects on grip (34.4–41.4%).

**Conclusion:**

A double dissociation emerged: Crus II GMV, but not dexterity, independently predicted grip strength, whereas dexterity, but not grip, predicted cognition. Grip strength and dexterity are therefore not interchangeable indices of motor function but map onto distinct substrates: posterior cerebellar integrity tracks maximal-force capacity, whereas fine motor performance indexes memory-related cognitive status independently of cerebellar volume.

## Introduction

Alzheimer’s disease (AD) is the leading cause of dementia, accounting for an estimated 60–80% of cases and producing characteristic neuropathological signatures including amyloid-*β* plaques, neurofibrillary tau tangles, synaptic dysfunction, and progressive neurodegeneration (McKhann et al. 2011; Alzheimer’s Association 2023; Jack et al. 2018). Although clinical dementia is most prevalent in older adulthood, the structural and molecular changes that underlie AD emerge decades before the onset of overt cognitive symptoms (Jack et al. 2018; Sperling et al. 2011), motivating a long-standing search for scalable early indicators of brain vulnerability and resilience across the adult lifespan. Established clinical instruments such as the Montreal Cognitive Assessment (MoCA) (Nasreddine et al. 2005) are well validated for detecting overt impairment but are insufficiently sensitive to the subtle cognitive shifts that characterize this preclinical window (Lindvall et al. 2024). Because conventional AD biomarker pipelines based on cerebrospinal fluid, positron emission tomography, and emerging plasma assays remain costly or require specialized infrastructure (Jack et al. 2018), there is increasing interest in functional phenotypes that can index brain integrity inexpensively and at population scale, with motor performance emerging as a particularly promising candidate that explains substantial variance in global cognition independent of demographic and health covariates (Schneider et al. 2025; Jost et al. 2025).

Within this functional-phenotype framework, grip strength has long been the most extensively studied indicator of physical reserve in aging. Hand-grip dynamometry predicts incident frailty, disability, and mortality in older adults (Bohannon 2008, 2019), and longitudinal evidence demonstrates a bidirectional coupling between grip strength and cognitive performance such that stronger grip predicts slower cognitive decline while higher cognition predicts better preserved grip (Cui et al. 2021; Cui et al. 2024). Frailty itself is increasingly conceptualized as a multidimensional syndrome in which sarcopenia, metabolic dysregulation, and cardiovascular processes jointly compromise nervous-system integrity (Jost et al. 2025; Anderer 2025). However, when grip strength is modeled alongside more cognitively demanding motor tasks, fine-motor measures such as the Nine-Hole Peg Test emerge as the dominant independent predictors of global cognition (Schneider et al. 2025), suggesting that more complex motor assessments may better capture the neural processes underlying age-related cognitive decline. Dexterity-based tasks appear particularly sensitive to cognitive load: dual-task paradigms demonstrate that adding a concurrent cognitive demand disproportionately degrades pegboard performance relative to other motor tasks (Petrigna et al. 2020), and visuospatial memory exerts measurable mediating effects on fine motor skill learning in older adults (Hooyman et al. 2023). Together, these findings raise the possibility that grip strength and dexterity, although routinely grouped under “motor function,” capture meaningfully different facets of central nervous system aging.

The cerebellum has emerged over the past three decades as a central structure that participates in both motor control and higher-order cognition. Once viewed as a purely motor structure, the cerebellum is now understood to support a broad range of functions including working memory, executive control, attention, language, and social cognition (Buckner 2013; Stoodley and Schmahmann 2009). This functional breadth reflects an anatomical division of labor within the cerebellum itself: anterior cerebellar lobules participate primarily in sensorimotor processing, whereas the posterior cerebellum, particularly the lateral hemispheres encompassing Crus I and Crus II, supports higher-order cognitive operations (Stoodley et al. 2012; Stoodley and Schmahmann 2009). The cerebellum is also among the structures most affected by normative aging, with substantial volumetric change observed across the adult lifespan (Ramanoël et al. 2018). Within this organization, grip strength and fine motor dexterity engage cerebellar circuitry in dissociable ways. Grip strength is determined by maximum voluntary force production and reflects a combination of central neural drive and substantial peripheral contributions including forearm musculature, neuromuscular junction integrity, and overall muscle mass (Bohannon 2019; Ward et al. 2007). In contrast, fine motor dexterity, as indexed by the Nine-Hole Peg Test, depends on the cerebellum’s contribution to error-based learning, online movement correction, and the temporal coordination of sequential finger movements (Lemon 2008; Stoodley et al. 2012). Because much of the existing literature has treated grip strength as a general proxy for neurological health without explicitly accounting for these peripheral contributions (Bohannon 2019), the more specific cerebellum–behavior associations afforded by fine motor measures may have been systematically obscured.

Among cerebellar regions, Crus I and Crus II warrant a particular *a priori* focus in the context of cognitive aging. As the supramodal zone of the posterior cerebellar hemisphere, Crus I and Crus II are anatomically and functionally distinguished from primary sensorimotor cerebellum (Buckner 2013; Stoodley et al. 2012; Stoodley and Schmahmann 2009). Structural aging studies have identified the right cerebellar Crus I/II region as showing reduced gray matter volume in older adults relative to younger comparison groups (Ramanoël et al. 2018), supporting its sensitivity to normative neural aging. Across the AD spectrum, voxel-based morphometry has revealed that Crus I atrophy emerges selectively at the AD dementia stage (Toniolo et al. 2018), while Crus II atrophy is detectable across the continuum from subjective cognitive decline through mild cognitive impairment (MCI) to AD dementia and tracks neuropsychological performance (Van Rossem et al. 2025). Cerebellar functional connectivity work converges on these regions, with progressive reductions in Crus I connectivity from MCI to AD tracking memory, executive function, visuospatial ability, and global cognition (Zhou et al. 2021), and disrupted Crus II–thalamic coupling linked to attention deficits in MCI and broader cognitive decline in AD (Tang et al. 2021). Causal animal work further confirms that Crus II neurons directly modulate recognition and spatial memory via a polysynaptic pathway through the cerebellar lateral nucleus and ventromedial thalamus (Fan et al. 2025). Together, this convergent structural, functional, and causal evidence positions Crus I and Crus II as cerebellar nodes that are particularly vulnerable in cognitive aging and well suited to interrogating brain–behavior relationships in mixed motor–cognitive constructs.

The present study therefore examined associations between grip strength, Nine-Hole Peg Test dexterity, cerebellar Crus I/II gray matter volume, and domain-specific cognitive performance assessed by the MoCA in a cross-sectional lifespan sample of healthy adults aged 20–79. We tested whether grip strength and dexterity dissociate in their cerebellar structural correlates and in their relationships to global cognition and individual MoCA subdomains, and used bootstrapped mediation analyses to evaluate whether dexterity and Crus II volume statistically account for portions of age-related variance in cognition and grip strength, respectively. By distinguishing the cerebellar and cognitive signatures of gross and fine motor performance, this work aims to clarify which motor measures are most informative for indexing cerebellar integrity in normative cognitive aging.

## Materials and Methods

### Participants

Data from the NIH Toolbox physiological measures were drawn from the University of South Carolina’s Aging Brain Cohort Study Repository, a multimodal lifespan database for studying the relationship between the brain, cognition, genetics, and behavior in healthy aging. As part of this study, T1-weighted magnetic resonance imaging (MRI) images were collected, and participants also completed grip strength and dexterity testing. Grip strength was tested using a Jamar Plus Dynamometer, with force recorded in pounds (lb), and dexterity was tested using the Nine-Hole Peg Test for both dominant and non-dominant hands. Grip strength and dexterity assessments were administered in accordance with standardized NIH Toolbox motor function protocols (National Institutes of Health Toolbox 2012). From September 2019 to December 2023, participants in the study underwent testing, filled out health-related forms and cognitive tests, and underwent an MRI scan using the University of South Carolina T1-weighted structural MRI scanner.

The University of South Carolina Institutional Review Board (IRB) approved this procedure. The 304 participants were between 20 and 79 years old (mean age = 46.74 years, SD = 19.38; 75 males, 229 females). By race, the sample comprised 251 White (82.6%), 32 Black or African American (10.5%), 12 Asian (3.9%), 8 Unknown or not reported (2.6%), and 1 participant reporting more than one race (0.3%). Eligibility for MRI scanning required participants to tolerate lying supine for approximately one hour, have a maximum girth below 60 inches, and weigh under 400 pounds. Participants did not have existing medical conditions including, but not limited to: severe illnesses such as cancer, untreated and unmanaged psychological conditions such as schizophrenia, and no current or past fatiguing illnesses. The sample comprised all participants in the Aging Brain Cohort repository meeting inclusion criteria for whom complete grip strength, dexterity, MoCA, and neuroimaging data were available. This dataset specified the sex, age, socioeconomic status, and race of each individual as fixed variables.

### Scanning Protocol

All participants underwent the same MRI scanning protocol at the University of South Carolina, McCausland Center for Brain Imaging on a Siemens Trio 3T scanner with a 20-channel head coil. T1-weighted images were used for volumetric analyses and were acquired using the following parameters: T1-weighted magnetization-prepared rapid gradient-echo (MP-RAGE) sequence with 1 mm isotropic voxels, 256×256 matrix size, 9° flip angle, and 192-slice sequence with repetition time = 2250 ms, inversion time = 925 ms, and echo time = 4.11 ms.

### Gray Matter Volume Extraction

**Figure 1:**
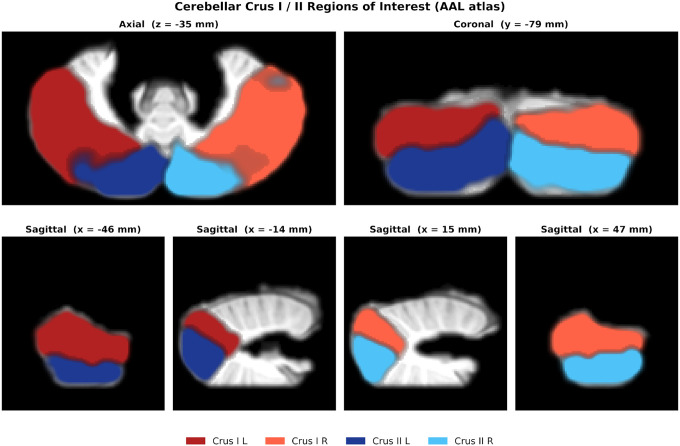
A priori cerebellar regions of interest (AAL3 atlas). Cerebellar Crus I (red shades) and Crus II (blue shades), left and right, rendered on the high-resolution MNI152 anatomical template. Top row: axial (z = −35 mm) and coronal (y = −79 mm) slices at the level of maximum combined ROI coverage. Bottom row: four sagittal slices (x = −46, −14, +15, +47 mm) stepping from lateral to medial across both hemispheres, illustrating the depth distribution of each Crus ROI. Non-cerebellar tissue is masked

To extract regional gray matter volumes, the Computational Anatomy Toolbox (CAT12) was employed with default settings (Gaser et al. 2024). CAT12 for voxel-based morphometry (VBM) employs a fully automated workflow that quantifies brain structure from MRI scans. The pipeline applies bias correction to minimize MRI artifacts, segments the brain into gray matter, white matter, and cerebrospinal fluid via tissue classification, spatially normalizes the images to a Montreal Neurological Institute (MNI) template, corrects for local brain size, and modulates the segments to preserve volume information. Cerebellar regions of interest (ROI) were defined using the Automated Anatomical Labeling 3 (AAL3) atlas (Rolls et al. 2020; Tzourio-Mazoyer et al. 2002) as bundled within the CAT12 pipeline. Four *a priori* regions of interest were extracted: left and right Crus I and left and right Crus II. To control for individual differences in head size, regional volumes were expressed as a proportion of each participant’s total intracranial volume (TIV). A volumetric rendering of the four ROIs on the MNI152 template is shown in Fig. 1.

### Statistical Methods

All statistical analyses were conducted in Python (version 3.13) using the pandas, NumPy, SciPy, and statsmodels libraries. Statistical significance was set at *α* = .05. Associations between variables were assessed using Pearson partial correlations, computed via an ordinary least squares (OLS) residualization approach: each variable of interest was regressed on the covariate set using OLS, and the Pearson correlation was then computed between the resulting residuals. Age was entered as a continuous covariate. Sex and race were treated as categorical variables and dummy-coded prior to entry, with sex = 1 (male) and race = 2 (White) serving as reference categories, yielding four dummy-coded race regressors and one dummy-coded sex regressor. This approach was used in preference to treating sex and race as continuous numeric codes, which would impose an unwarranted ordinal structure on nominal variables.

False discovery rate (FDR) correction was applied using the Benjamini–Hochberg procedure. For Crus I/II (L and R) × motor measure associations (dominant and non-dominant, grip strength and dexterity), correction was applied across the full family of 16 tests (four ROIs × four motor outcomes). For dexterity × MoCA associations, correction was applied across eight tests (dominant dexterity × MoCA total score and seven index subscores). Non-dominant dexterity and grip strength × MoCA associations were examined separately and evaluated at uncorrected *α* = .05 given their exploratory nature; no associations in these families survived even uncorrected thresholds. Two-tailed *p*-values were used for all FDR corrections.

Bootstrapped mediation analyses were conducted to test indirect effects of age on grip strength (via Crus II gray matter volume) and on MoCA total score (via dominant hand dexterity). For each model, age served as the independent variable (*X*), the proposed mediator (*M*) was either Crus II volume or dexterity, and the dependent variable (*Y*) was either grip strength or MoCA total score. Sex and race, coded nominally, were entered as covariates in all paths. Path coefficients were estimated using OLS regression. Indirect effects (*a*×*b*) were computed as the product of the path *a* coefficient (*X*→*M*, controlling for covariates) and the path *b* coefficient (*M*→*Y*, controlling for *X* and covariates). Statistical significance of indirect effects was evaluated using 5,000 bootstrap iterations with percentile 95% confidence intervals (CI); an indirect effect was considered significant if the confidence interval excluded zero. The proportion of the total effect mediated was computed as the indirect effect divided by the total effect (*c* path).

To formally evaluate the proposed double dissociation, the strengths of competing overlapping partial correlations were compared using the procedure of Meng et al. (1992) for dependent correlations that share a common variable. Two planned contrasts were tested: whether left Crus II volume was more strongly associated with grip strength than with dexterity, and whether MoCA total score was more strongly associated with dexterity than with grip strength. For these comparisons, motor measures were sign-aligned so that higher values denoted better performance, and age, sex, and race were partialled from all variables before comparison; two-tailed p-values are reported.

Analyses were conducted on all available complete cases for each specific analysis, with listwise deletion applied per model. Sample sizes ranged from 297 to 304 across analyses, reflecting missing values in grip strength (*n* = 5–6 missing) and dexterity (*n* = 6–7 missing) assessments. With a sample of approximately 300 participants, the present study had approximately 80% power to detect partial correlations of *r*≥.16 (two-tailed, *α* = .05).

## Results

The final analytic sample comprised 304 participants (75 male, 229 female; mean age = 46.74 ± 19.38 years, range: 20–79). Descriptive statistics for all primary variables are presented in Table 1.

**Figure 2:**
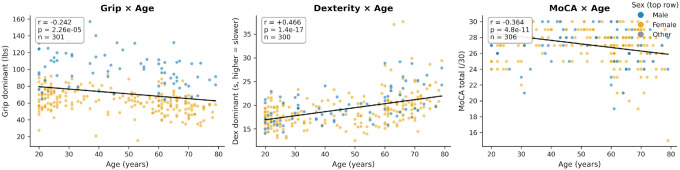
Grip strength, dexterity, and cognition as a function of age. Scatterplots of dominant grip strength (lb), dominant Nine-Hole Peg Test time (s; higher = slower), and MoCA total score against age, with points colored by sex. Bivariate Pearson correlations, p-values, and n are inset. Grip strength declines and peg time increases with age, whereas MoCA scores are comparatively stable

**Figure 3:**
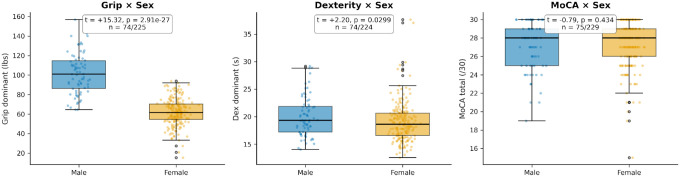
Distributions of grip strength, dexterity, and cognition by sex. Box-and-jitter plots of dominant grip strength (lb), dominant peg time (s), and MoCA total score for male and female participants. Independent-samples t statistics, p-values, and group sizes are inset. Grip strength differs markedly by sex; dexterity differs modestly; MoCA does not

**Figure 4:**
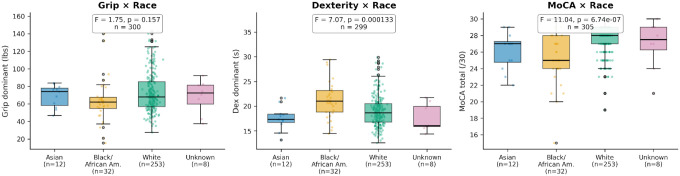
Distributions of grip strength, dexterity, and cognition by race. Box-and-jitter plots of dominant grip strength (lb), dominant peg time (s), and MoCA total score across racial groups (Asian, Black/African American, White, Unknown). One-way analysis of variance (ANOVA) F statistics, p-values, and group sizes are inset. The single participant reporting more than one race is omitted from these panels

The bivariate distributions of grip strength, dexterity, and MoCA across age, sex, and race are shown in Figs. 2–4. Grip strength and dexterity both varied with age, and grip strength differed markedly by sex, whereas MoCA scores were comparatively stable across demographic groups.

### Relationships between Grip Strength and Dexterity

First, the study sought to understand the relationship between grip strength and dexterity. All four grip strength × dexterity pairings across dominant and non-dominant hands were significantly negatively correlated after controlling for age, sex, and race (dominant grip × dominant dexterity: *r*=−.241, *p*<.001; dominant grip × non-dominant dexterity: *r*=−.154, *p*=.008; non-dominant grip × dominant dexterity: *r*=−.154, *p*=.008; non-dominant grip × non-dominant dexterity: *r*=−.168, *p*=.004; all *n* = 296–299). Higher grip strength was consistently associated with faster dexterity, confirming that the two measures are related but not redundant motor constructs.

### Cerebellar Crus I/II Volumes and Grip Strength

Having established that grip strength and dexterity represent related but distinct motor constructs, the next aim was to determine whether variation in these behaviors corresponded to gray matter volume differences within *a priori* cerebellar regions of interest. Partial correlations between cerebellar Crus I and Crus II gray matter volumes and grip strength were computed with age, sex, and race entered as covariates. FDR correction was applied across all 16 Crus I/II × motor measure associations.

Cerebellar Crus II volume was positively associated with grip strength following FDR correction. Crus II left correlated significantly with non-dominant grip strength (*r*=.224, *p*_FDR_=.002, *n* = 298) and dominant grip strength (*r*=.156, *p*_FDR_=.038, *n* = 299). Crus II right correlated significantly with non-dominant grip strength (*r*=.198, *p*_FDR_=.005, *n* = 298). In all cases, larger Crus II volume was associated with stronger grip. Cerebellar Crus I showed numerical trends in the same direction for non-dominant grip (Crus I left: *r*=.140, *p*_FDR_=.063; Crus I right: *r*=.131, *p*_FDR_=.077) that did not survive FDR correction. Full results are presented in Table 2.

**Figure 5:**
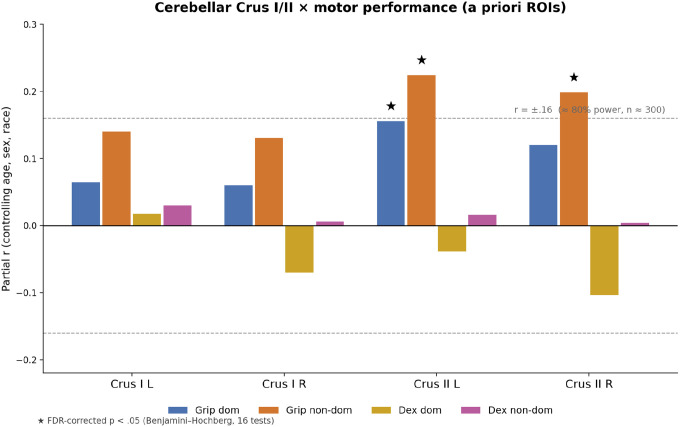
Partial correlations between cerebellar Crus I/II volume and motor performance. Partial correlations (controlling for age, sex, and race) between left and right Crus I and Crus II gray matter volume and four motor outcomes: dominant and non-dominant grip strength and dominant and non-dominant dexterity. The dashed reference line marks r = ±.16 (≈80% power at n ≈ 300). Asterisks mark associations surviving Benjamini–Hochberg FDR correction across the 16-test family (* FDR-corrected p < .05)

**Figure 6:**
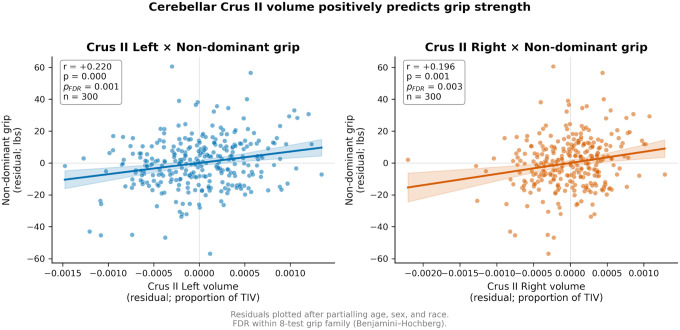
Cerebellar Crus II volume positively predicts non-dominant grip strength. Residual scatterplots of non-dominant grip strength against left and right Crus II gray matter volume, after partialling age, sex, and race from both variables. Partial r, uncorrected and FDR-corrected p-values, and n are inset; shaded bands are 95% confidence intervals. The two strongest FDR-significant Crus II–grip associations are shown here; the third FDR-significant association (left Crus II with dominant grip) is reported in Table 2. FDR was applied within the 16-test grip family (Benjamini–Hochberg)

The full set of Crus I/II × motor partial correlations is displayed in Fig. 5, and the Crus II–grip relationships that survived FDR correction are shown as residual scatterplots in Fig. 6.

### Cerebellar Crus I/II Volumes and Dexterity

After comparing grip strength correlations to selected ROI GMV, dexterity was then correlated with the same ROIs. No significant associations were observed between cerebellar Crus I or Crus II volumes and Nine-Hole Peg Test dexterity performance after FDR correction (all *p*_FDR_>.16). The largest observed uncorrected association was *r*=−.10 (Crus II right × dominant dexterity, *p*=.073, *p*_FDR_=.168). With the present sample size (*n* ≈ 300), the study was adequately powered at approximately 80% to detect correlations of *r*≥.16 (two-tailed, *α* = .05), indicating that these results constitute evidence against moderate-to-large associations rather than a failure of statistical power.

### Dexterity, Grip Strength, and Cognitive Performance

**Figure 7:**
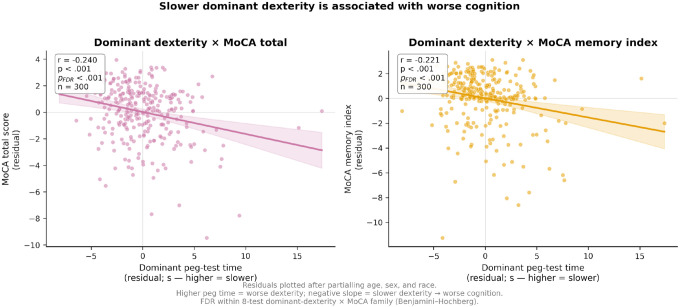
Slower dominant-hand dexterity is associated with poorer cognition. Residual scatterplots of MoCA total score (left) and MoCA memory index (right) against dominant Nine-Hole Peg Test time, after partialling age, sex, and race. Higher peg time indicates slower (worse) dexterity. Partial r, uncorrected and FDR-corrected p-values, and n are inset; shaded bands are 95% confidence intervals. FDR was applied across the eight-test dexterity – MoCA family (Benjamini–Hochberg)

To further evaluate the clinical relevance of motor performance, the next study aim was to determine whether grip strength and dexterity were viable markers of cognition. Partial correlations between motor measures and MoCA total/subscores (memory, executive, visuospatial, orientation, language, attention/concentration, and immediate recall) were conducted controlling for age, sex, and race, with FDR correction applied across eight dexterity × MoCA comparisons. Dominant hand dexterity was significantly associated with MoCA total score (*r*=−.240, *p*_FDR_<.001, *n* = 298) and the MoCA memory index (*r*=−.220, *p*_FDR_<.001, *n* = 298; slower peg time = lower MoCA performance). No other MoCA subscores survived FDR correction (executive index: *r*=−.110, *p*_FDR_=.157; all others *p*_FDR_>.16). Non-dominant dexterity did not survive FDR correction for MoCA total or any MoCA subscore. Grip strength, whether dominant or non-dominant, was not significantly associated with any MoCA subscore after FDR correction (all *p*_uncorr_>.07). These dexterity–cognition associations are shown in Fig. 7.

### Mediation of Age Effects on Cognition Through Dexterity

**Figure 8:**
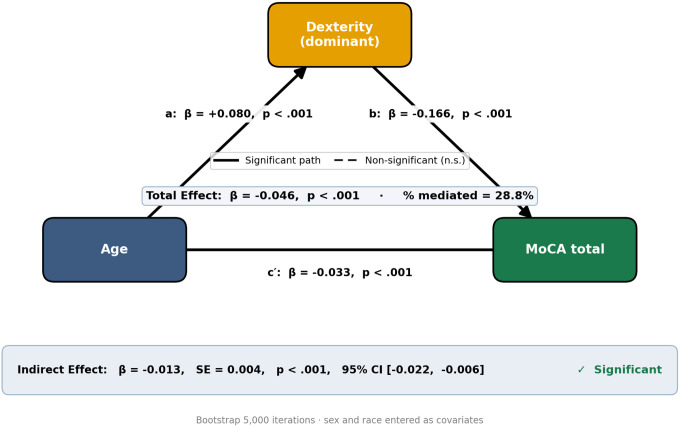
Dominant-hand dexterity partially mediates the effect of age on cognition. Mediation model with age as the independent variable, dominant Nine-Hole Peg Test dexterity as the mediator, and MoCA total score as the outcome; sex and race entered as covariates. Path coefficients (a, b, c, c′) with standard errors and p-values are shown, together with the bootstrapped indirect effect (5,000 iterations, 95% CI) and the proportion of the total effect mediated. Solid arrows denote significant paths

Because dominant hand dexterity was associated with both aging and cognitive performance, mediation analyses were conducted to test whether dexterity statistically accounted for part of the age-related decline in cognition. A bootstrapped mediation analysis (5,000 iterations) examined whether dominant hand dexterity mediated the association between age and MoCA total score, with sex and race entered as covariates. Age significantly predicted dexterity performance (path *a*: *b* = 0.080, standard error [SE] = 0.010, *p*<.001), indicating that older age was associated with slower peg completion times. Dexterity in turn significantly predicted MoCA score (path *b*: *b*=−0.166, SE = 0.039, *p*<.001). The total effect of age on MoCA was significant (path *c*: *b*=−0.046, SE = 0.007, *p*<.001) and remained significant after accounting for dexterity (direct effect *c*^’^: *b*=−0.033, SE = 0.007, *p*<.001), indicating partial mediation. The indirect effect was statistically significant (*a*×*b*=−0.013, SE_BOOT_=0.004, 95% CI [−0.022,−0.006]), with dexterity accounting for approximately 28.8% of the total effect of age on MoCA performance. This mediation model is illustrated in Fig. 8.

### Mediation of Age Effects on Grip Strength Through Cerebellar Crus II Volume

Given that age was associated with reduced grip strength and that Crus II volume correlated with grip performance, additional mediation analyses tested whether age-related reductions in cerebellar volume partially explained declines in grip strength. Parallel bootstrapped mediation analyses (5,000 iterations) tested whether Crus II gray matter volume mediated the relationship between age and grip strength, with sex and race as covariates. Older age was significantly associated with smaller Crus II volume (Crus II left, path *a*: *b*=−1.40×10^− 5^, SE = 1.52×10^− 6^, *p*<.001; Crus II right, path *a*: *b*=−1.19×10^− 5^, SE = 1.38×10^− 6^, *p*<.001). Larger Crus II volume was in turn significantly associated with stronger non-dominant grip strength (Crus II left, path *b*: partial *r*=.224, *p*<.001; Crus II right, path *b*: partial *r*=.198, *p*<.001).

Indirect effects through Crus II volume were significant for non-dominant grip: through Crus II left (−0.102, 95% CI [−0.157,−0.048]), accounting for approximately 41.4% of the total age effect on non-dominant grip; and through Crus II right (−0.085, 95% CI [−0.140,−0.037]), accounting for approximately 34.4%. Significant indirect effects were also observed for dominant grip through Crus II left (−0.064, 95% CI [−0.114,−0.017], 23.3% mediated) and Crus II right (−0.047, 95% CI [−0.095,−0.006], 16.8% mediated). In all models, total and direct effects of age on grip strength remained significant, confirming partial mediation. Mediation results are summarized in Table 3. The two dominant-grip indirect effects (via left and right Crus II) are reported for completeness but are best regarded as exploratory, because the corresponding Crus II-dominant-grip partial correlations did not survive FDR correction (Table 2).

**Figure 9:**
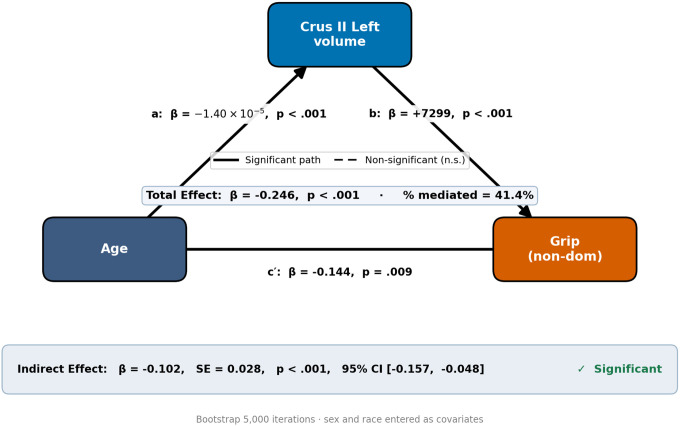
Left Crus II gray matter volume partially mediates the effect of age on non-dominant grip strength. Mediation model with age as the independent variable, left Crus II volume as the mediator, and non-dominant grip strength as the outcome; sex and race entered as covariates. Path coefficients with standard errors and p-values are shown, together with the bootstrapped indirect effect (5,000 iterations, 95% CI) and the proportion of the total effect mediated. Solid arrows denote significant paths

The Crus II left → non-dominant grip mediation model is illustrated in Fig. 9.

### Formal Test of the Double Dissociation

The hypothesized double dissociation was supported by direct comparison of the competing partial correlations (Table 4). Left Crus II volume was associated with non-dominant grip strength significantly more strongly than with dominant dexterity (Z = 2.33, p = .020) and with non-dominant dexterity (Z = 3.17, p = .002). Conversely, MoCA total score was associated with dominant dexterity significantly more strongly than with dominant grip strength (Z = 2.86, p = .004) and than with non-dominant grip strength (Z = 2.35, p = .019). The corresponding right Crus II contrast (grip versus dexterity) was in the same direction but did not reach significance (Z = 1.08, p = .280), consistent with the left-lateralized emphasis of the primary findings.

## Discussion

The present study examined associations between cerebellar Crus I/II gray matter volume, grip strength, manual dexterity, and cognitive performance in 304 healthy adults, controlling for age, sex, and race. Three primary findings emerged. First, bilateral Crus II volume was robustly associated with grip strength and partially mediated the age–grip relationship. Second, neither Crus I nor Crus II volume was significantly associated with dexterity, despite adequate power to detect moderate effects. Third, dominant hand dexterity was selectively associated with MoCA total score and the memory index and partially mediated the age–cognition relationship. Grip strength and dexterity, though related, thus have dissociable cerebellar and cognitive correlates: posterior cerebellar structural integrity indexes grip capacity, whereas dexterity captures cognitive aging independently of Crus I/II volume.

The finding that bilateral Crus II volume correlates positively with grip strength, surviving FDR correction with partial correlations ranging from *r*=.156 to *r*=.224, represents the primary structural finding of this study. Mediation further revealed that age-related reduction in Crus II volume accounts for 34.4–41.4% of the total effect of age on non-dominant grip strength. These results align with converging evidence implicating Crus I and Crus II in higher-order functions and identifying posterior cerebellar atrophy as a sensitive indicator of neural aging (Ramanoël et al. 2018; Van Rossem et al. 2025). The present data extend these observations to grip strength, identifying Crus II as a cerebellar marker of grip capacity.

The grip–Crus II association was most consistent for non-dominant grip across both hemispheres. One interpretation is that non-dominant grip, being less influenced by task-specific practice and habitual hand use, may more directly index central cerebellar integrity than dominant grip, which is more strongly shaped by peripheral neuromuscular factors and lateralized skill. This interpretation is speculative and warrants longitudinal or intervention-based study. Crus I showed numerical trends in the same direction as Crus II that did not survive FDR correction, consistent with the two regions occupying adjacent positions within the cognitive cerebellum but possibly reflecting greater statistical sensitivity for Crus II in the present sample.

The Crus II–grip association was not paralleled by a Crus II–dexterity association. No Crus I or Crus II region showed a significant relationship with Nine-Hole Peg Test performance after FDR correction, with the largest uncorrected effect being *r*=−.10. The present study was powered to detect correlations of *r*≥.16 at 80%, meaning that moderate-to-large effects of Crus I/II on dexterity can be excluded with reasonable confidence, though small effects below this threshold cannot be ruled out. This dissociation suggests that Crus II volume specifically reflects the motor capacity component captured by grip strength rather than the fine coordinated movement captured by dexterity.

This dissociation was statistically reliable, not an artifact of comparing a significant with a non-significant correlation: left Crus II volume was associated with grip significantly more strongly than with dexterity, and MoCA with dexterity significantly more strongly than with grip (all p < .05; Table 4). Its direction may initially seem counterintuitive, because Crus I/II are usually framed as cognitive regions and dexterity is the more cognitively demanding task. Two considerations help reconcile this. First, Crus II volume is a slowly accruing structural index of neural integrity, whereas Nine-Hole Peg Test performance is state-sensitive, shaped by attentional, peripheral, and practice factors; volume may therefore track the stable, capacity-like component that grip also reflects, rather than the dynamic online-control demands of pegboard performance. Second, the grip–Crus II association need not imply that Crus II executes gross movement: posterior cerebellar volume covaries with global brain and neuromuscular aging, so it may index a whole-organism integrity expressed more faithfully in maximal force output than in a brief, ceiling-prone dexterity task. Dexterity thus carries cognitive information that is not localized to Crus I/II volume, as evidenced by its selective link to the MoCA memory index, whereas Crus II volume indexes a strength-relevant integrity that is cognitively non-specific. These interpretations are post hoc and should be tested with task-based functional imaging and longitudinal designs.

Dominant hand dexterity was selectively associated with MoCA total score and the memory index following FDR correction, with no other subscores surviving. These findings are consistent with evidence that dexterity-based measures are sensitive, independent predictors of cognition in older adults (Schneider et al. 2025), and extend that work by demonstrating selectivity for the memory domain, namely delayed recall and recognition, which are among the domains most sensitive to early neurodegenerative change. Grip strength was not significantly associated with any MoCA subscore, consistent with the hypothesis that dexterity, rather than gross strength, is the more sensitive motor correlate of cognitive status.

Dexterity partially mediated the association between age and MoCA total score, with the indirect effect accounting for 28.8% of age’s total effect. The direct effect remained significant after entering dexterity, confirming partial rather than full mediation, consistent with age-related cognitive decline proceeding via multiple pathways, of which declining fine motor performance is one.

### Atlas Choice and Cerebellar Parcellation

The present analyses used the Automated Anatomical Labeling 3 (AAL3) atlas (Rolls et al. 2020; Tzourio-Mazoyer et al. 2002) for cerebellar parcellation, as bundled within the CAT12 VBM pipeline (Gaser et al. 2024). We acknowledge that the Spatially Unbiased Infratentorial Template (SUIT) cerebellar atlas (Diedrichsen et al. 2009) provides higher-resolution lobular boundaries and is widely used in cerebellum-focused research. AAL3 was selected for three reasons. First, its Crus I and Crus II are anatomically defined whole-lobule units whose boundaries correspond well to the same-named SUIT lobules. Second, our *a priori* hypotheses concerned whole-lobule volumes rather than subregional gradients. Third, the AAL family is among the most widely deployed atlases in neuroaging and dementia VBM, affording direct comparability with prior cerebellar studies. We regard AAL3 as a conservative choice prioritizing comparability and reproducibility; replication in SUIT-parcellated data is recommended.

### Limitations and Future Studies

Several limitations must be acknowledged. First, the cross-sectional design precludes causal inference; all associations and mediation pathways described here are correlational. Second, MoCA total scores exhibit a pronounced ceiling effect in this cognitively healthy sample, which likely attenuated cognitive associations. Third, the sample was drawn from a single academic medical center, limiting demographic and geographic generalizability. Fourth, while the study was adequately powered to detect moderate effects (*r*≥.16), small true effects for Crus I/II and dexterity cannot be excluded. Fifth, the sample comprised substantially more female (*n* = 229) than male (*n* = 75) participants, limiting the generalizability of findings to men. Finally, all neuroimaging data were acquired at a single time point, precluding assessment of trajectory-based associations.

## Conclusion

This study provides cross-sectional evidence for a double dissociation in the cerebellar and cognitive correlates of grip strength and manual dexterity. Cerebellar Crus II volume is robustly and bilaterally associated with grip strength, surviving FDR correction, and partially mediates the effect of age on grip. In contrast, manual dexterity is selectively linked to MoCA total score and the memory index, also FDR-robust, and partially mediates the age–cognition relationship, while showing no significant structural association with cerebellar Crus I/II. These findings underscore the importance of differentiating motor measures in the study of cerebellum–behavior relationships in aging: grip strength and dexterity are related motor constructs with distinct cerebellar and cognitive signatures, and treating them as interchangeable risks obscuring both the cerebellar structural correlates of strength and the cognitive relevance of fine motor coordination.

## Figures and Tables

**Table 1:** Descriptive statistics for demographic, motor, cognitive, and cerebellar volume measures. Sample size (N), mean, standard deviation (SD), minimum, and maximum for each measure (N = 304). Grip strength is in pounds (lb); dexterity is Nine-Hole Peg Test completion time in seconds (s). Cerebellar Crus I and Crus II gray matter volumes (left and right) are expressed as a proportion of total intracranial volume. N varies across measures owing to missing grip strength and dexterity assessments.

Variable	N	Mean	SD	Min	Max
Age (years)	304	46.74	19.38	20.00	79.00
Grip strength − dominant (lb)	299	71.83	23.07	15.40	156.90
Grip strength − non-dominant (lb)	298	68.03	23.87	17.69	161.80
Dexterity − dominant (s)	298	19.24	3.56	12.55	37.66
Dexterity − non-dominant (s)	297	20.07	3.12	12.48	31.78
MoCA total	304	27.33	2.40	15.00	30.00
Crus I − left	304	0.0068	0.0007	0.0043	0.0086
Crus I − right	304	0.0059	0.0007	0.0018	0.0076
Crus II − left	304	0.0051	0.0006	0.0034	0.0068
Crus II − right	304	0.0045	0.0005	0.0020	0.0061

**Table 2:** Partial correlations between cerebellar Crus I/II gray matter volume and grip strength. Partial correlation coefficient (r), uncorrected p-value, Benjamini–Hochberg FDR-corrected p-value, and sample size (n) for each cerebellar region of interest by grip strength outcome, controlling for age, sex, and race. Rows are ordered by effect size. FDR correction was applied across the full family of 16 Crus I/II by motor associations. Asterisks (*) denote associations surviving FDR correction (FDR-corrected p < .05).

ROI	Outcome	r	p (uncorr.)	p (FDR)	n
Crus II − left	Grip non-dominant	.224	< .001	.002*	298
Crus II − right	Grip non-dominant	.198	.001	.005*	298
Crus II − left	Grip dominant	.156	.007	.038*	299
Crus I − left	Grip non-dominant	.140	.016	.063	298
Crus I − right	Grip non-dominant	.131	.024	.077	298
Crus II − right	Grip dominant	.120	.038	.102	299
Crus I − left	Grip dominant	.065	.266	.472	299
Crus I − right	Grip dominant	.060	.302	.483	299

**Table 3:** Bootstrapped mediation of age effects on grip strength and cognition. Indirect effects, 95% percentile confidence intervals (5,000 bootstrap iterations), and proportion of the total age effect mediated, for each mediator → outcome pathway. Sex and race were entered as covariates in all paths. An indirect effect was considered significant when its 95% CI excluded zero.

Mediator	Outcome	Indirect effect	95% CI	% mediated
Crus II left	Grip non-dominant	−0.102	[− 0.157, − 0.048]	41.4%
Crus II right	Grip non-dominant	−0.085	[− 0.140, − 0.037]	34.4%
Crus II left	Grip dominant	−0.064	[− 0.114, − 0.017]	23.3%
Crus II right	Grip dominant	−0.047	[− 0.095, − 0.006]	16.8%
Dexterity (dom.)	MoCA total	−0.013	[− 0.022, − 0.006]	28.8%

**Table 4:** Formal tests of the double dissociation (Meng et al. 1992 comparison of overlapping partial correlations). In each contrast the two partial correlations share a common variable (left or right Crus II volume, or MoCA total score). The r values are partial correlations controlling for age, sex, and race, with motor measures sign-aligned so that higher denotes better performance; Z and the two-tailed p-value test whether the two correlations differ. Rows 1–4 establish the dissociation for left Crus II and for cognition; the right Crus II contrast (row 5) is directional but non-significant. n = 296–297 per contrast.

Shared variable	Comparison (stronger vs weaker)	r (stronger)	r (weaker)	Z	p
Left Crus II	grip (non-dom) vs dexterity (dom)	.223	.046	2.33	.020
Left Crus II	grip (non-dom) vs dexterity (non-dom)	.223	−.016	3.17	.002
MoCA total	dexterity (dom) vs grip (dom)	.240	.035	2.86	.004
MoCA total	dexterity (dom) vs grip (non-dom)	.227	.049	2.35	.019
Right Crus II	grip (non-dom) vs dexterity (dom)	.196	.115	1.08	.280

## Data Availability

Data were drawn from the University of South Carolina Aging Brain Cohort Study Repository. The datasets generated and/or analyzed during the current study are available from the author upon request.

## Code availability

All code used is publicly available or available upon request.

## References

[R1] Alzheimer’s Association (2023) 2023 Alzheimer’s disease facts and figures. Alzheimers Dement 19:1598–1695. 10.1002/alz.13016 36918389

[R2] Anderer S (2025) Frailty symptoms associated with increased dementia risk. JAMA 334:1506. 10.1001/jama.2025.17486 41042505

[R3] Bohannon RW (2008) Hand-grip dynamometry predicts future outcomes in aging adults. J Geriatr Phys Ther 31:3–10. 10.1519/00139143-200831010-00002 18489802

[R4] Bohannon RW (2019) Grip strength: an indispensable biomarker for older adults. Clin Interv Aging 14:1681–1691. 10.2147/CIA.S194543 31631989 PMC6778477

[R5] Buckner RL (2013) The cerebellum and cognitive function: 25 years of insight from anatomy and neuroimaging. Neuron 80:807–815. 10.1016/j.neuron.2013.10.044 24183029

[R6] Cui M, Zhang S, Liu Y, Gang X, Wang G (2021) Grip strength and the risk of cognitive decline and dementia: a systematic review and meta-analysis of longitudinal cohort studies. Front Aging Neurosci 13:625551. 10.3389/fnagi.2021.625551 33613270 PMC7890203

[R7] Cui M, Wang J, Deng M (2024) Longitudinal relationship between grip strength and cognitive function in a European population older than 50 years: a cross-lagged panel model. Arch Gerontol Geriatr 122:105396. 10.1016/j.archger.2024.105396 38484671

[R8] Diedrichsen J, Balsters JH, Flavell J, Cussans E, Ramnani N (2009) A probabilistic MR atlas of the human cerebellum. NeuroImage 46:39–46. 10.1016/j.neuroimage.2009.01.045 19457380

[R9] Fan Y, Tian M, Chen Y (2025) Cerebellar Crus II regulates recognition and spatial memory in mice. Mol Neurobiol 62:10320–10332. 10.1007/s12035-025-04852-2 40198447

[R10] Gaser C, Dahnke R, Thompson PM, Kurth F, Luders E, Alzheimer’s Disease Neuroimaging Initiative (2024) CAT: a computational anatomy toolbox for the analysis of structural MRI data. Gigascience 13:giae049. 10.1093/gigascience/giae049 39102518 PMC11299546

[R11] Hooyman A, Lingo VanGilder J, Schaefer SY (2023) Mediation analysis of the effect of visuospatial memory on motor skill learning in older adults. J Mot Behav 55:68–77. 10.1080/00222895.2022.2105793 35902117 PMC9792432

[R12] Jack CR, Bennett DA, Blennow K (2018) NIA-AA research framework: toward a biological definition of Alzheimer’s disease. Alzheimers Dement 14:535–562. 10.1016/j.jalz.2018.02.018 29653606 PMC5958625

[R13] Jost Z, Kujach S (2025) Understanding cognitive decline in aging: mechanisms and mitigation strategies – a narrative review. Clin Interv Aging 20:459–469. 10.2147/CIA.S510670 40256418 PMC12009036

[R14] Lemon RN (2008) Descending pathways in motor control. Annu Rev Neurosci 31:195–218. 10.1146/annurev.neuro.31.060407.125547 18558853

[R15] Lindvall E, Abzhandadze T, Quinn TJ, Sunnerhagen KS, Lundström E (2024) Is the difference real, is the difference relevant: the minimal detectable and clinically important changes in the Montreal Cognitive Assessment. Cereb Circ Cogn Behav 6:100222. 10.1016/j.cccb.2024.100222 38745691 PMC11090903

[R16] McKhann GM, Knopman DS, Chertkow H (2011) The diagnosis of dementia due to Alzheimer’s disease: recommendations from the National Institute on Aging-Alzheimer’s Association workgroups on diagnostic guidelines for Alzheimer’s disease. Alzheimers Dement 7:263–269. 10.1016/j.jalz.2011.03.005 21514250 PMC3312024

[R17] Meng X-L, Rosenthal R, Rubin DB (1992) Comparing correlated correlation coefficients. Psychol Bull 111:172–175. 10.1037/0033-2909.111.1.172

[R18] Nasreddine ZS, Phillips NA, Bédirian V (2005) The Montreal Cognitive Assessment, MoCA: a brief screening tool for mild cognitive impairment. J Am Geriatr Soc 53:695–699. 10.1111/j.1532-5415.2005.53221.x 15817019

[R19] National Institutes of Health Toolbox (2012) NIH Toolbox training manual. Northwestern University and the National Institutes of Health

[R20] Petrigna L, Pajaujiene S, Iacona GM (2020) The execution of the Grooved Pegboard Test in a dual-task situation: a pilot study. Heliyon 6:e04678. 10.1016/j.heliyon.2020.e04678 32817897 PMC7426567

[R21] Ramanoël S, Hoyau E, Kauffmann L (2018) Gray matter volume and cognitive performance during normal aging: a voxel-based morphometry study. Front Aging Neurosci 10:235. 10.3389/fnagi.2018.00235 30123123 PMC6085481

[R22] Rolls ET, Huang C-C, Lin C-P, Feng J, Joliot M (2020) Automated anatomical labelling atlas 3. NeuroImage 206:116189. 10.1016/j.neuroimage.2019.116189 31521825

[R23] Schneider TR, Felbecker A, von Mitzlaff B (2025) Hand dexterity and mobility independently predict cognition in older adults: a multi-domain regression analysis. Front Aging Neurosci 17:1624307. 10.3389/fnagi.2025.1624307 40933827 PMC12417501

[R24] Sperling RA, Aisen PS, Beckett LA (2011) Toward defining the preclinical stages of Alzheimer’s disease: recommendations from the National Institute on Aging-Alzheimer’s Association workgroups. Alzheimers Dement 7:280–292. 10.1016/j.jalz.2011.03.003 21514248 PMC3220946

[R25] Stoodley CJ, Schmahmann JD (2009) Functional topography in the human cerebellum: a meta-analysis of neuroimaging studies. NeuroImage 44:489–501. 10.1016/j.neuroimage.2008.08.039 18835452

[R26] Stoodley CJ, Valera EM, Schmahmann JD (2012) Functional topography of the cerebellum for motor and cognitive tasks: an fMRI study. NeuroImage 59:1560–1570. 10.1016/j.neuroimage.2011.08.065 21907811 PMC3230671

[R27] Tang F, Zhu D, Ma W, Yao Q, Li Q, Shi J (2021) Differences in cerebellar functional connectivity between mild cognitive impairment and Alzheimer’s disease: a seed-based approach. Front Neurol 12:645171. 10.3389/fneur.2021.645171 34220669 PMC8248670

[R28] Toniolo S, Serra L, Olivito G, Marra C, Bozzali M, Cercignani M (2018) Patterns of cerebellar gray matter atrophy across Alzheimer’s disease progression. Front Cell Neurosci 12:430. 10.3389/fncel.2018.00430 30515080 PMC6255820

[R29] Tzourio-Mazoyer N, Landeau B, Papathanassiou D (2002) Automated anatomical labeling of activations in SPM using a macroscopic anatomical parcellation of the MNI MRI single-subject brain. NeuroImage 15:273–289. 10.1006/nimg.2001.0978 11771995

[R30] Van Rossem D, Allemeersch G-J, Barros N (2025) Assessing the link between cerebellar volume and cognitive function in Alzheimer’s disease: a pilot study. Sci Rep 15:32943. 10.1038/s41598-025-12975-8 41006361 PMC12475151

[R31] Ward NS, Newton JM, Swayne OBC (2007) The relationship between brain activity and peak grip force is modulated by corticospinal system integrity after subcortical stroke. Eur J Neurosci 25:1865–1873. 10.1111/j.1460-9568.2007.05434.x 17432972 PMC3715370

[R32] Zhou Z, Zhu R, Shao W (2021) Changes in resting-state functional connectivity of cerebellum in amnestic mild cognitive impairment and Alzheimer’s disease: a case-control study. Front Syst Neurosci 15:596221. 10.3389/fnsys.2021.596221 33790747 PMC8006280

